# Vascular Endothelial Growth Factor-A Deficiency in Perivascular Adipose Tissue Impairs Macrovascular Function

**DOI:** 10.3389/fphys.2019.00687

**Published:** 2019-06-14

**Authors:** Raffi Gharakhanian, Shi Su, Tamar Aprahamian

**Affiliations:** ^1^Division of Graduate Medical Sciences, Boston University School of Medicine, Boston, MA, United States; ^2^Renal Section, Department of Medicine, Boston University School of Medicine, Boston, MA, United States; ^3^The Center for Metabolic Health, Boston Medical Center, Boston, MA, United States

**Keywords:** angiogenesis, perivascular, brown adipose tissue, macrovascular, VEGF, endothelium

## Abstract

**Objective:** Thoracic perivascular adipose tissue (PVAT) has been shown to release factors that influence the functioning of neighboring vascular tissue. Cardiovascular complications of obesity are on the rise; therefore, this study set out to determine if adipose-specific ablation of vascular endothelial growth factor-A (VEGF-A) plays a role in the maintenance of aortic structure and function.

**Methods:** Adipose-specific VEGF-A-deficient mice were previously generated. Fabp4cre(+). VEGF*^flox/flox^* and Fabp4cre(−). VEGF*^flox/flox^* mice were maintained on chow diet. PVAT gene expression was measured with real-time quantitative PCR. Aortic vasomotor response was assessed with isometric tension measurements. Collagen deposition was analyzed histologically in the vascular media and compared using ratiometric pigment density.

**Results:** PVAT-specific adiponectin expression was decreased in Fabp4cre(+). VEGF*^flox/flox^* mice. Isometric tension measurements revealed a dose-dependent dysfunction in response to acetylcholine within the distal aortic segment of Fabp4cre(+). VEGF*^flox/flox^*. Fabp4cre(+). VEGF*^flox/flox^* mice exhibited increased aortic deposition of collagen within the thoracic adventitial and medial spaces.

**Conclusion:** These data demonstrate that decreased expression of VEGF-A within the surrounding adipose tissue microenvironment of the thoracic aorta has a detrimental effect on aortic integrity and vascular function. Modulation of angiogenic pathways within PVAT may offer an important avenue toward the treatment of adipose tissue dysfunction in obesity and its vascular complications.

## Introduction

Arterial stiffening is a loss of arterial elasticity, progressing with age (Bramwell and Hill, 1922; Hallock, 1934; Eliakim et al., 1971; Tounian et al., 2001; Mitchell et al., 2004; McEniery et al., 2005) and obesity (Sutton-Tyrrell et al., 2001; Safar et al., 2006), therefore affecting a large portion of the population. It is a precursor to isolated systolic hypertension (Najjar et al., 2008; Kaess et al., 2012), which increases the risk of stroke and heart disease (Nielsen et al., 1995; Blacher et al., 1999; Meaume et al., 2001; Boutouyrie et al., 2002; Cruickshank et al., 2002). With a large majority of the United States population expected to be obese by 2030, it is critical to understand the mechanisms by which increased adiposity or changes to adipose tissue composition contribute to vascular dysfunction.

The presence of metabolically active brown adipose tissue (BAT) in human adults has been confirmed (Nedergaard et al., 2010). The quantity of BAT is also negatively correlated with obesity and age (Yoneshiro et al., 2011); in addition, imaging studies show metabolically active BAT along the thoracic, but not the abdominal, spinal area (Cypess et al., 2015). This adipose tissue surrounding the thoracic aorta is similar to interscapular BAT in structure and gene expression pattern (Fitzgibbons et al., 2011). Because of its juxtaposition, thoracic perivascular adipose tissue (PVAT) influences vascular function, mainly by releasing vasoactive, and in the context of obesity, inflammatory molecules (Soltis and Cassis, 1991; Löhn et al., 2002; Verlohren et al., 2004; Gao et al., 2009; Greenstein et al., 2009). High-cholesterol and high-fat diet increase PVAT pro-inflammatory cytokine production in mouse models of atherosclerosis and abdominal aortic aneurysm respectively, further linking obesity to increased risk of cardiovascular disease (Police et al., 2009; Dobrian et al., 2015). In addition, decreased expression of adiponectin, an anti-inflammatory cytokine released by adipocytes, is observed in PVAT under conditions of obesity. Local administration of recombinant adiponectin in mice is able to reverse neointimal thickening observed in obesity (Takaoka et al., 2009), providing evidence that functional adipokines are released by PVAT. However, it should be noted that these studies were performed on adipose tissue depots that are phenotypically similar to white adipose tissue (WAT). The detailed examination of the paracrine function of perivascular brown adipocytes is warranted and important, as it will provide novel targets to modulate cardiovascular pathologies associated with both aging and obesity.

VEGF is a pro-angiogenic molecule known to stimulate vasodilation (Horowitz et al., 1997), while VEGF neutralization is associated with hypertension (Hurwitz et al., 2004; Levine et al., 2004). It is widely known that VEGF produced by smooth muscle cells (SMCs) (Williams et al., 1995) acts through its receptors on endothelial cells to regulate normal and pathological angiogenesis (Ferrara and Davis-Smyth, 1997). VEGF receptors are also expressed on SMCs (Brown et al., 1997; Couper et al., 1997; Wang and Keiser, 1998; Grosskreutz et al., 1999), and their activation critically regulates atherosclerosis and neointimal hyperplasia. Mouse models with overexpression of VEGF in adipose tissue demonstrate increased vascularization within adipose tissue, as well as, protection against systemic metabolic dysfunction (as induced by high-fat diet) (Elias et al., 2012; Sun et al., 2012, 2014). It has been shown that deletion of VEGF in adipose tissue results in increased WAT inflammation, BAT lipid accumulation, and disturbances with glucose tolerance (Sung et al., 2013; Shimizu et al., 2014); however, the role of endogenous VEGF in PVAT function and SMC regulation were not addressed. Therefore, determining the effect of brown adipocyte-derived VEGF on PVAT function, vascular tone, and cardiovascular disease related to aging and obesity has become increasingly important.

Although the capacity of the angiogenic signal provided by VEGF has been shown to drive BAT development (Sun et al., 2014), the critical role of brown adipocyte-derived VEGF in maintaining SMC function has not been demonstrated. Correlative studies using conditioned media demonstrate increased VEGF release from visceral and epicardial PVAT of obese and diabetic patients which induces vascular SMC proliferation (Schlich et al., 2013), however these studies do not assess the mechanical properties of the SMC (Qiu et al., 2010).

## Materials and Methods

### Animals

Fourteen week-old-male mice with adipose-specific VEGF-A ablation were previously generated (Mahdaviani et al., 2017) using (Fabp4-cre #005069, Jackson Labs) and VEGF*^flox/flox^* mice (provided by Genentech, Inc.). Fabp4cre(−) VEGF*^flox/flox^* (*n* = 4) and Fabp4cre(+) VEGF*^flox/flox^* (*n* = 8) mice were euthanized and PVAT dissected from a portion of the thoracic aorta was snap frozen in liquid nitrogen for RNA analysis. Another portion of the thoracic aorta was left intact and fixed in 10% formalin and processed for histological analysis. The Institutional Animal Care and Use Committee of Boston University School of Medicine approved these experiments.

### Real-Time Quantitative PCR

Tissue from PVAT and BAT were processed using a Qiagen system. Tissue was disrupted with a TissueLyser and RNA extracted using the Qiagen RNeasy kit (Valencia, CA) followed by transcription using a QuantiTect® reverse transcription kit. Gene expression was analyzed using a ViiA™ 7 RealTime PCR system. Taqman Gene Expression Assays were performed using primer sets: Ucp1(#Mm01244861_m) and Adiponectin to analyzed transcript levels relative to GAPDH (#Mm99999915_m). mRNA quantitation was calculated and expressed as fold change relative to control tissue.

### VEGF-A Protein Quantification

Tissue from PVAT, BAT, and WAT samples were collected and processed by a TissueLyser (Qiagen) and protein extraction was carried out. VEGF-A quantification was determined using a mouse VEGF-A ELISA kit (R&D systems).

### Isometric Tension Measurement

Thoracic aortas were collected and placed in a chilled physiological salt solution (PSS) (KCl 4.7 mM, CaCl_2_ 2.5 mM, KH_2_PO_4_ 1.2 mM, MgSO_4_ 0.6 mM, NaHCO_3_ 25 mM, NaCl 118.3 mM, and dextrose 5.5 mM). The PVAT was carefully removed and snap frozen in liquid nitrogen for RNA analysis. Each aorta was subsequently cut into 3–4 mm sections starting 1 mm distal to the left subclavian artery branch from the aortic arch. Aortic rings were hung in a constantly aerated bath of PSS then incrementally stretched to 2 g of tension. Rings were then tested for maximum contraction and viability with 10 min of 50 mM KCl. Washout was performed with PSS, then preconstricted to 70–80% of maximum with phenylephrine using half-log cumulative concentrations from 10^−8^–10^−5^ mol/L. Relaxation was performed with cumulative concentrations of Ach from 10^−8^–10^−5^ mol/L. Isometric tension analysis was performed as previously described (Adachi and Cohen, 2000).

### Histological Characterization of Aorta

The aortas were perfused with 10% formalin to maintain a patent lumen. The aortas were then removed with PVAT attached, fixed overnight, and then prepared for paraffin embedding. 5-μm specimens were cut and stained using Masson’s trichrome (Sigma-Aldrich HT15-KT). The level of collagen deposition, indicated by an Aniline blue stain, was analyzed using Photoshop CS6 as a percentage of the aortic wall.

### Statistical Analysis

Data are presented as mean ± SEM and a Student’s two tailed *t*-test was used for comparison between groups. *p* < 0.05 is considered statistically significant.

## Results

We recently published that the vast majority of VEGF in adipose tissue is derived from the adipocyte precursor fraction, with minimal expression of VEGF in other cell populations. As previously shown, and reproduced here, the level of VEGF-A in the BAT is three-fold greater than that of WAT (Mahdaviani et al., 2016). Since mitochondria play an important role in thermogenesis and brown adipocyte function, we hypothesized that the lack of VEGF was responsible for our observations. We differentiated preadipocytes from interscapular BAT of Fabp4cre(+). VEGF^flox/flox^ and control mice. Protein expression levels of VEGF, UCP1, and porin (VDAC) (used to asses mitochondrial content), and norepinephrine-stimulated uncoupling were all decreased in the adipocytes lacking VEGF. Acute ablation of VEGF by adenoviral-Cre knockdown resulted in decreased VEGF without affecting porin levels and decreased response to norepinephrine albeit to a lesser extent than chronic conditions (Mahdaviani et al., 2016).

### VEGF Is Required for Brown Adipocyte Formation and Maintenance

**Figure 1:**
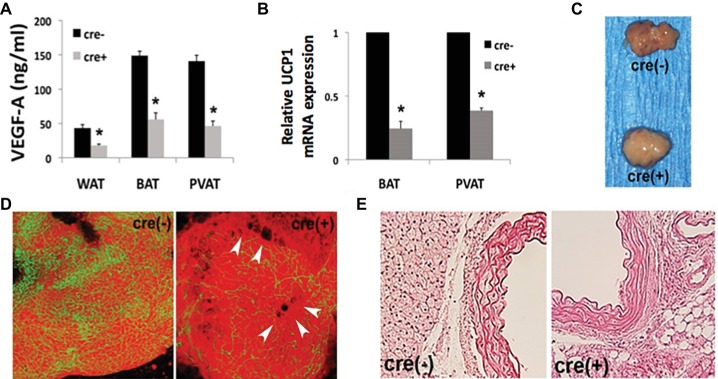
Disrupted adipose tissue phenotype in Fabp4cre(+). VEGF*^flox/flox^* mice. **(A)** Vascular endothelial growth factor-A (VEGF-A) ablation results in decreased VEGF-A protein expression measured by ELISA in white adipose tissue (WAT), brown adipose tissue (BAT), and perivascular adipose tissue (PVAT). **(B)** RT-PCR of PVAT from male Fabp4cre(+). VEGF*^flox/flox^* mice (*n* = 8) is reported as a fold-change relative to Fabp4cre(−) VEGF*^flox/flox^* (*n* = 4) control mice, normalized to GAPDH. **(C)** Gross examination of dissected thoracic PVAT. **(D)** Confocal micrographs depicting vascularity of thoracic PVAT [visualized by BS1-lectin (FITC) for vasculature and BODIPY (Texas Red) for adipocytes] and lipid droplet coalescence (arrowheads) (50 μm stack). **(E)** Representative photomicrographs of thoracic stained with hematoxylin and eosin (40× mag.). *p < 0.05.

Since thoracic PVAT is molecularly similar to interscapular BAT (Fitzgibbons et al., 2011) and we find a strong BAT-associated phenotype in our mouse model, we performed an in-depth analysis of PVAT and vascular function. We show a significant reduction of VEGF protein in all adipose tissue depots, with a concomitant reduction in UCP1 expression in BAT and PVAT from mice harboring the cre-transgene (Figures 1A,B). Gross examination reveals “whitened” thoracic PVAT in the absence of VEGF (Figure 1C). Confocal microscopy shows decreased PVAT vascularization near areas of lipid droplet coalescence in Fabp4cre(+). VEGF^flox/flox^ mice versus controls (Figure 1D). Morphological analysis reveals enlarged lipid droplets in the absence of VEGF (Figure 1E).

### Brown Adipocyte-Derived VEGF Is Required for Aortic Remodeling

**Figure 2:**
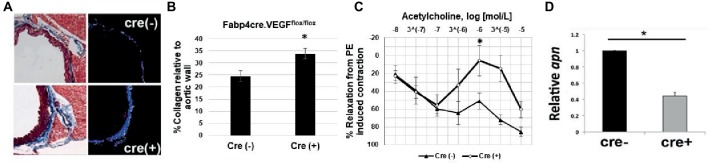
Impaired vascular tone in Fabp4cre(+). VEGF*^flox/flox^* mice. **(A)** Representative photomicrographs stained with Masson’s Trichrome (left) and area of analysis (right). **(B)** Quantification of collagen deposition in Fabp4cre(+). VEGF*^flox/flox^* (*n* = 4) and Fabp4cre(−). VEGF*^flox/flox^* (*n* = 4) groups. **(C)** Fabp4cre(+). VEGF*^flox/flox^* mice ACh-induced relaxation response is impaired in thoracic aorta. ACh response is expressed as % relaxation of a PE-induced contraction. Ring relaxation response cre (+) (*n* = 8) and cre (−) (*n* = 4). **(D)** Decreased adiponectin (apn) in PVAT of Fabp4cre(+). VEGF^flox/flox^ mice (*n* = 4). *p < 0.05.

To assess extracellular remodeling, and potential increased risk toward the development of arterial stiffness, due to adipose-specific VEGF deficiency, thoracic aorta samples were collected from young male Fabp4cre(±). VEGF*^flox/flox^* mice maintained on chow diet. Collagen levels were analyzed in the medial spaces of the aorta by first determining the number of pixels that constituted the vascular media then subtracting the number of Aniline blue stained collagen pixels (Figure 2A). Significantly increased collagen deposition was observed within the aortic media and adventitia of VEGF-deficient mice [cre(+) 33.82 ± 2.15% vs. cre(−) 24.25 ± 2.24%] (Figure 2B).

### VEGF-A Deletion in UCP1^+^ Cells Results in Impaired Aortic Relaxation

We hypothesized that the decreased vascularity within adipose tissue will lead to impaired vasomotor function, which is a prognostic indicator of vascular dysfunction. Functional relevance is evidenced by impaired acetylcholine (ACh)-induced aortic relaxation in Fabp4cre(+). VEGF^flox/flox^ mice compared to controls (Figure 2C). Adiponectin is predominantly secreted from adipose tissue and its function to induce smooth muscle relaxation has been shown to be an important mechanism for vascular function and homeostasis (Lynch et al., 2013). We observed a significant decrease in adiponectin expression in Fabp4cre(+). VEGF*^flox/flox^* PVAT compared to Fabp4cre(−).VEGF*^flox/flox^* (Figure 2D).

## Discussion

The present study was designed to elucidate the effects of capillary rarefaction in PVAT and the subsequent impact on macrovascular function. Here, we demonstrate that PVAT from Fabp4cre(+). VEGF*^flox/flox^* show a decrease in vascularity and increased lipid coalescence. The results of this study demonstrate that VEGF-A deficiency in PVAT is a contributing factor toward several elements of vascular dysfunction. We found that our Fabp4cre(+). VEGF*^flox/flox^* model displayed increased collagen deposition and decreased aortic relaxation in the thoracic aorta in young mice on chow diet, suggesting that there is an early physiological response to decreased VEGF-A within the PVAT.

Paradoxically, the concentration of adiponectin in circulation is inversely proportional to adipose mass, suggesting that adipose quality rather than quantity is more important for expression of adiponectin (Turer et al., 2011). In terms of the anti-fibrotic role of adiponectin, this inverse correlation is also observed, with reduced circulating concentrations associated with increased fibrosis (Marangoni et al., 2017). Although adiponectin expression in PVAT has been previously described (Antoniades et al., 2009), we further demonstrated that VEGF ablation affected the levels of adiponectin expressed in the PVAT. While this decreased adiponectin expression in PVAT may play an important role in this model, we cannot exclude the fact that other adipokines may be contributing to vascular dysfunction.

Endothelial cells are a dynamic paracrine organ and play a crucial role in maintaining vascular tone and mediating inflammatory processes. Endothelial dysfunction from obesity-induced metabolic syndrome is characterized by a decrease in nitric oxide (NO) production (Calles-Escandon and Cipolla, 2001). Our isometric tension measurements revealed that Fabp4cre(+). VEGF*^flox/flox^* aortic rings exhibited a dysfunctional vasomotor response which suggests that VEGF-A deficiency has both a structural and biochemical influence.

Limitations of our study include the possibility that the observed effects are attributed solely to PVAT. As such, the lack of PVAT during aortic ring isometric tension studies prevents conclusively determining the vasomodulatory properties of PVAT. To account for any changes occurring specifically by thoracic PVAT in our model, future studies may include isometric tension experiments with preserved PVAT and denervation of BAT. In addition, we acknowledge that we cannot exclude the possibility that the microvasculature is contributing to any observed effects and would hypothesize that small vessels may also be damaged in the long term, should hypertension be observed. Furthermore, since it is possible that there may be two arms to VEGF signaling – angiogenic and metabolic – one could envision devising a biased agonist to selectively activate the metabolic arm, thereby improving PVAT function without inducing a deleterious angiogenic response.

Taken together, these experiments suggest that the microvascular environment of PVAT has an important effect on the function of its surrounding tissue, specifically, the ability of endothelial cells to modulate vascular tone. With the increasing prevalence of obesity and its association with capillary rarefaction in adipose tissue, further exploring the role of PVAT within this dynamic system is warranted. Such studies can help provide greater insight to various other comorbidities such as hypertension.

Our *in vivo* data suggest that VEGF produced by adipocytes cells prevents collagen accumulation in the aortic wall, and *ex vivo* experiments demonstrate a requirement of adipose-derived VEGF in aortic relaxation. Our data also show that impaired VEGF signaling by adipocytes leads to abnormal aortic tone, remodeling, and stiffness, suggesting that paracrine effects of VEGF from adipocytes in the thoracic PVAT maintains adipose tissue and could contribute to effects on vascular integrity. Therefore, cardiovascular complications, as observed in obesity and aging, may benefit from modulation of this critical pathway to improve disease conditions.

## Data Availability

The raw data supporting the conclusions of this manuscript will be made available by the authors, without undue reservation, to any qualified researcher.

## Ethics Statement

The Institutional Animal Care and Use Committee of Boston University School of Medicine approved these experiments.

## Author Contributions

RG and TA designed the study, performed experiments, and wrote the paper. SS provided technical assistance with experiments.

### Conflict of Interest Statement

The authors declare that the research was conducted in the absence of any commercial or financial relationships that could be construed as a potential conflict of interest.
